# Efficiency of Different Superplasticizers and Retarders on Properties of ‘One-Part’ Fly Ash-Slag Blended Geopolymers with Different Activators

**DOI:** 10.3390/ma12203410

**Published:** 2019-10-18

**Authors:** Shin Hau Bong, Behzad Nematollahi, Ali Nazari, Ming Xia, Jay Sanjayan

**Affiliations:** Centre for Smart Infrastructure and Digital Construction, Faculty of Science, Engineering and Technology, Swinburne University of Technology, Melbourne, Victoria 3122, Australia; sbong@swin.edu.au (S.H.B.); alinazari@swin.edu.au (A.N.); mxia@swin.edu.au (M.X.); jsanjayan@swin.edu.au (J.S.)

**Keywords:** retarder, superplasticizer, one-part geopolymer, just-add-water, setting time, workability, compressive strength

## Abstract

Currently, there are a very limited number of studies on the effect of admixtures on properties of ‘one-part’ geopolymers. This paper reports the effects of different superplasticizers and retarders on fresh and hardened properties of one-part fly ash-slag blended geopolymers made by different solid activators. Two different grades of sodium silicate, namely anhydrous sodium metasilicate powder (nSiO_2_/nNa_2_O = 0.9) and GD Grade sodium silicate powder (nSiO_2_/nNa_2_O = 2.0) were used as the solid activators. Five different commercially available superplasticizers, including three modified polycarboxylate-based superplasticizers (denoted as PC1, PC2, and PC3) and two naphthalene-based superplasticizers (denoted as N1 and N2), as well as three different retarders, including sucrose, anhydrous borax and a commercially available retarder, were investigated. Workability, setting time and compressive strength of the mixtures without and with addition of each ‘individual’ admixture were measured. The results showed the effect of admixtures on the properties of the one-part geopolymers significantly depended on the type of solid activator and the type of admixture used. When GD Grade sodium silicate powder was used as the solid activator, all investigated admixtures not only had no positive effect on the workability and setting time, but also significantly reduced the compressive strength of the mixture. However, when anhydrous sodium metasilicate powder was used as the solid activator, the PC1 and sucrose were the best performing superplasticizer and retarder, respectively, causing no reduction in the compressive strength, but significant increase in the workability (up to + 72%) and setting time (up to + 111%), respectively as compared to the mixture with no admixture. In addition, the results also showed that addition of ‘*combined*’ admixtures (i.e., PC1 in the presence of sucrose) significantly increased the workability (up to + 39%) and setting time (up to + 141%), but slightly reduced the compressive strength (−16%) of the mixture activated by anhydrous sodium metasilicate powder, as compared to the mixture with no admixture.

## 1. Introduction

Ordinary Portland cement (OPC) has been widely used as the main binder for production of concrete in the construction industry. However, the manufacture of OPC contributes approximately 5–7% of the total global CO_2_ emissions [1]. Moreover, the production of OPC is an energy-intensive activity; 3–7 GJ of energy is needed to manufacture one ton of OPC [2]. Hence, it is essential to develop alternative OPC-less binders for concrete production. Geopolymer is an alternative binder to OPC, which can be manufactured from materials of geological origin (such as metakaolin) or industrial by-products (such as fly ash and slag that contain a considerable amount of silica and alumina) with high alkaline activators [3]. The utilization of industrial by-products such as fly ash as the source material in geopolymer production is particularly beneficial as disposition of these industrial by-products occupies large areas in landfills, which could be used for other purposes [4]. According to previous studies, using fly ash as the source material for production of geopolymer consumes up to 60% less energy and emits up to 80% less carbon dioxide as compared to production of OPC [5,6]. It is worth noting that the environmental footprint of geopolymer considerably depends on the source material and the activator used for its production. Besides the environmental advantages, geopolymers can exhibit a variety of properties and characteristics which are desirable in the construction industry, including high compressive strength, good chemical resistance, adjustable setting time, low shrinkage and excellent fire resistance [7,8,9,10,11].

Although geopolymer can offer the above-mentioned remarkable properties; however, the use of geopolymer, as compared to OPC, has so far been limited to small-scale projects and niche applications. This is mainly attributed to two important obstacles. The first obstacle is the use of user-hostile alkaline solutions to make geopolymers. Conventional geopolymer is synthesized from a ‘two-part’ mix formulation, which consists of liquid activators and solid aluminosilicate source materials. One of the important drawbacks of the ‘two-part’ mix formulation is that handling of large amount of corrosive and often viscous alkaline solutions is often difficult and challenging in commercial and large-scale applications of geopolymers [3]. The second obstacle is that geopolymers, especially low-calcium (Class F) fly ash-based mixtures, often require to be cured at elevated temperature to obtain better microstructural development and mechanical properties [12,13,14]. The necessity for the heat curing does not only limit its in-situ and large-scale applications, but also requires extra cost and energy associated with it, which limits commercial viability of geopolymer.

Several researchers have attempted to tackle both of the aforementioned obstacles by developing ambient temperature cured ‘one-part’ fly ash-slag blended geopolymers [3,9,15,16,17,18,19,20,21]. Such geopolymers are synthesized from a ‘just-add-water’ mix formulation, where only water is added to a pre-mixed dry material. In the pre-mixed dry material, a small amount of solid activator is used instead of large quantities of alkaline solution, and some percentages of fly ash are replaced with slag. However, the resulting geopolymer concrete often exhibit poor workability and short setting time [9,22], which may limit its commercial viability and large-scale applications. Therefore, it is essential to increase workability and setting time of ambient temperature cured ‘one-part’ fly ash-slag blended geopolymer concretes to enhance their commercial viability and large-scale applications.

High range water reducers, also known as superplasticizers (SPs), are often added to OPC-based concrete to improve its rheology, workability and mechanical properties [23,24,25]. It is a common practice to use SPs to improve the workability of OPC-based concrete with lower water content and achieve higher strength and durability. There are several types of SPs commercially available, including lignosulphonates, naphthalene-based, melamine-based, and modified polycarboxylates [25]. In the last few decades, the effect and mechanism of SPs in OPC have been widely studied by many researchers [26,27,28,29,30,31]. Although SPs were developed and optimized to work in OPC-based mixtures, several studies have investigated the efficiency of SPs in conventional ‘two-part’ geopolymer-based mixtures. It was found that SPs in general performed poorly in geopolymer systems due to instability of SP under highly alkaline environment [24,25,32,33,34,35]. However, studies on the effect of commercial SPs on properties of ‘one-part’ geopolymers are very limited. For instance, Luukkonen et al. [36] recently investigated the effect of different SPs on properties of a one-part geopolymer mortar made of slag as the source material, in conjunction of microsilica as an additional silica source, and sodium hydroxide powder as the solid activator. The authors concluded that for the investigated one-part geopolymer formulation lignosulfonate, melamine, and naphthalene-based SPs were more effective than polyacrylate and polycarboxylate-based SPs. Alrefaei et al. [37] investigated the effect of different SPs in a one-part geopolymer made of fly ash (50% *w*/*w*) and slag (50% *w*/*w*) as the source material and sodium silicate powder (8% *w*/*w*) as the solid activator with different water contents. They found that polycarboxylate-based SP was more effective for mixtures with high water content (i.e., water/precursor ≥ 0.36), while naphthalene-based SP was more effective for mixtures with low water content (i.e., water/precursor ≤ 0.36). 

Retarders (RTs) are often added to OPC-based concrete to increase its setting time [38,39,40,41,42,43]. Similar to SPs, RTs were also developed to work in OPC-based system. Previous studies have investigated the efficiency of RTs in conventional ‘two-part’ geopolymer mixtures. For instance, Kusbiantoro et al. [44] studied the effect of sucrose and citric acid on fly ash-based geopolymers. They reported that sucrose had retarding effect, while citric acid exhibited accelerating effect. In addition, they also reported that the compressive strength of geopolymer decreased by increasing dosage of sucrose and citric acid. Liu et al. [45] investigated the influence of anhydrous borax on the setting mechanism of fly ash-based geopolymer at elevated temperature. They concluded that anhydrous borax only exhibited the retarding effect when its dosage reached to a certain value. In addition, they also found that the compressive strength of specimens with anhydrous borax was significantly decreased. Garg and White [46] reported that zinc oxide was very effective in retarding the slag-based geopolymer. Other materials such as sodium phosphate [47], phosphoric acid [48], sodium chloride and malic acid [49] were also studied by other researchers as RTs for geopolymers. However, studies on the effect of retarders on properties of one-part geopolymers are very limited. Recently, Oderji et al. [50] investigated the effect of different RTs on a one-part geopolymer composed of fly ash (85% *w*/*w*), slag (15% *w*/*w*), and sodium silicate powder (12% *w*/*w*). Among the admixtures investigated in their study, it was reported that borax was the most effective one when both compressive strength and workability of the mixture was concerned.

Based on the above review, further research on effect of SPs and RTs on properties of one-part geopolymers is necessary. This is because the available results are not yet conclusive about the influences of type and dosage of admixtures used, as well as type of solid activators and source materials, along with time and temperature of curing used for production of one-part geopolymers. Therefore, this study aims to investigate the influence of different commercially available SPs and RTs on both fresh and hardened properties of one-part geopolymer pastes made of fly ash and slag as the source materials and two different grades of sodium silicate powders with different alkali modulus as the solid activators. In addition, it is a common practice to use more than one type of admixture (e.g., a RT in the presence of a SP) to obtain desirable rheological properties of OPC-based concrete. However, the effect of combined admixtures on one-part geopolymers has rarely been studied. Therefore, this study also investigates the effects of ‘combined’ admixtures (i.e., addition of the best performing SP in the presence of the best performing RT) on properties of the ‘one-part’ fly ash-slag blended geopolymers.

## 2. Materials and Methods 

### 2.1. Geopolymer Precursors

A low-calcium (Class F) fly ash and ground granulated blast furnace slag were used as the aluminosilicate source materials in this study. The fly ash was supplied by Cement Australia Pty Ltd., Brisbane, Australia. The slag was supplied by Building Products Supplies Pty Ltd., Melbourne, Australia. SPECTRO XEPOS X-ray Fluorescence (XRF) spectrometer (SPECTRO Analytical Instruments Inc., Mahwah, NJ, USA) was used to determine the chemical composition of the fly ash and slag used in this study. Table 1 presents the results of XRF analysis.

### 2.2. Solid Activators

Two types of sodium silicate powders, namely anhydrous sodium metasilicate and GD Grade sodium silicate were used as the solid activators in this study. The anhydrous sodium metasilicate and GD Grade sodium silicate powders were supplied by Redox Pty Ltd., Melbourne, Australia and PQ Australia Pty Ltd., Melbourne, Australia, respectively. The specifications of different types of sodium silicate powders are presented in Table 2.

### 2.3. Admixtures

Five different SPs including three modified polycarboxylate (denoted as PC1, PC2 and PC3) and two naphthalene-based SPs (denoted as N1 and N2) were investigated in this study. These SPs are commercially available products in Australia supplied by BASF (Melbourne) and SIKA (Melbourne). The specifications of the SPs investigated in this paper are presented in Table 3.

Three types of RTs were used in this paper. Two of them were the proposed RTs for geopolymer-based systems including sucrose and anhydrous borax (henceforth referred to as borax) powders. The sucrose (denoted as S) and borax (denoted as B) were supplied by Chem-Supply Pty Ltd., Adelaide, Australia and IMCD Australia Limited, Melbourne, Australia, respectively. A commercially available retarder for OPC concrete (denoted as RT) supplied by BASF, Melbourne, Australia was also investigated in this paper. The specifications of the RTs investigated in this paper are presented in Table 4.

## 3. Experimental Procedures

The experimental program performed was divided into two parts (Part I and Part II). In Part I, different SPs and RTs were used *separately* to study the effects of addition of an *individual admixture (i.e., a SP or a RT)* on properties of one-part fly ash-slag blended geopolymer made by different activators. The best performing SP and RT exhibiting desirable properties were selected based on the results obtained in Part I. In Part II, the best performing SP and RT were used *together* to study the effects of addition of *combined admixtures (i.e., the best performing SP in the presence of the best performing RT)* on properties of the one-part geopolymer.

### 3.1. Mixture Proportions

In this paper, two series of geopolymer mixtures were prepared based on the type of activator, namely AN series (which use anhydrous sodium metasilicate powder as the activator) and GD series (which use GD Grade sodium silicate powder as the activator). Each series comprised of nine mixtures as shown in Table 5. In all mixtures, the mass ratio of fly ash to slag was kept constant equal to 1.0. The mass ratio of solid activator to geopolymer precursors (fly ash + slag) was also kept constant equal to 0.1. The water-to-geopolymer-solids ratio (W/GP-solids) of each mixture as defined by Hardjito et al. [51] was also kept constant equal to 0.367. The mass ratio of water to geopolymer precursors (fly ash + slag) in AN series mixtures was equal to 0.4. In order to keep the W/GP-solids constant in all mixtures, the mass ratio of water to geopolymer precursors in GD series mixtures was adjusted (equal to 0.379) to correspond to the same W/GP-solids of 0.367. The mass ratio of SPs or RTs to geopolymer precursors in each mixture was kept constant equal to 0.01.

### 3.2. Mixing, Casting, Curing and Testing of Specimens

To prepare the mixtures, fly ash, slag, solid activator and retarder (if any) were added to a 20-littre Hobart mixer and dry mixed at low-speed for three min. Tap water was then slowly added to the mixture and the mixture was mixed for another two min at low-speed. Subsequently, the SP (if any) was added to the mixer and the mixture was mixed for another eight min at high-speed. The workability of each mixture was measured immediately after completion of the mixing process by conducting a mini-slump test in accordance with ASTM C1437 [52]. The initial and final setting times of each mixture were measured by using a Vicat apparatus according to AS/NZS 2350.4 [53]. The setting time was measured from the time that the water was added to the dry materials. Six 25 mm cube specimens were cast from each mixture for compression tests. The specimens were placed in a sealed container and put in an oven at 60 °C for 24 h. After the end of heat curing period, the specimens were removed from the oven and left in the laboratory environment to cool down and then removed from the molds. After de-molding, the specimens were stored at ambient temperature (23 ± 3°C) until the day of testing. Previous studies reported that heat-cured geopolymers typically reach more than 90% of their long-term strength after three days [3,17,18]. Therefore, all specimens were tested three days after casting in this study. According to AS 1012.9 [54], compression tests were conducted under load control at the rate of 20 ± 2 MPa/min. 

## 4. Results and Discussions

### 4.1. Effects of Different Superplasticisers

#### 4.1.1. Workability

**Figure 1:**
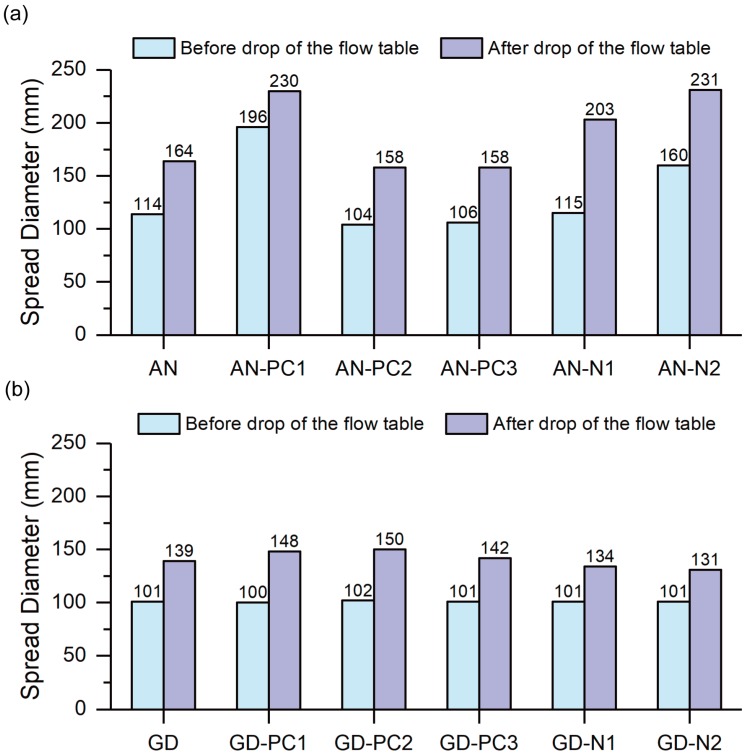
Effect of different SPs on workability of one-part fly ash-slag blended geopolymer pastes activated by (**a**) Anhydrous sodium metasilicate powder, and (**b**) GD Grade sodium silicate powder.

The average spread diameter of each mixture without and with different SPs is presented in Figure 1. By comparing the mixtures without using any SP, the “AN” mix exhibited larger spread diameters both before and after drop of the flow table as compared to “GD” mix. This is despite the fact that both of these mixtures had identical W/GP-solids, as shown in Table 5. Previous studies reported that slump or slump flow is affected by yield stress of the paste [55,56]. Therefore, the higher workability of “AN” paste indicates its lower yield stress than that of “GD” paste.

As can be seen in Figure 1a, the spread diameters of “AN-PC1” and “AN-N2” pastes (both before and after drop of the flow table) were considerably increased with respect to those of the paste without any SP (“AN” paste). As compared to “AN” paste, the increase in spread diameter of “AN-PC1” and “AN-N2” pastes before drop of the flow table were 72% and 40%, respectively and after drop of the flow table were 40% and 41%, respectively. Although the average spread diameters of “AN-N1” and “AN” pastes before drop of the flow table were comparable; however, the spread diameter of “AN-N1” paste after drop of the flow table was 24% larger than that of “AN” paste. On the other hand, the spread diameters of “AN-PC2” and “AN-PC3” pastes before and after drop of the flow table were 4–9% smaller as compared to those of “AN” paste.

Based on the aforementioned results, it can be concluded that among the SPs investigated in this study, PC1, N2 and N1 were chemically stable (to some extent) in the anhydrous sodium metasilicate solution, thus provided up to 40% increase in workability of the geopolymer paste [32,57]. However, PC2 and PC3 were chemically unstable (i.e., experienced structural changes) in the anhydrous sodium metasilicate solution, and thereby lost their superplasticizing properties.

It is interesting to note that the PC1 supplied by BASF Australia was effective in increasing the workability of the geopolymer paste, but PC2 and PC3 supplied by SIKA Australia were not effective. Considering that all these SPs (PC1, PC2 and PC3) were PCE-based, therefore it can be said that not all types of PCE-based SPs undergo structural changes in the anhydrous sodium metasilicate solution. Palacios and Puertas [32,57] also stated that not all types of SPs undergo structural changes in highly alkaline media and the structural degradation depends on the type of SP and the type of alkaline solution used. However, due to unavailability of technical data (such as molecular weight, chemical structure, additives, etc.) for these *commercial* superplasticisers, it is not possible to investigate the cause of stability of PC1 versus instability of PC2 and PC3 in the anhydrous sodium metasilicate solution.

On the contrary, as can be seen in Figure 1b, in case of the mixtures activated with GD Grade sodium silicate powder, the average spread diameter of all pastes with and without SP before drop of the flow table was almost 100 mm, which is equal to the bottom diameter of the mini-slump cone used in this paper. After drop of the flow table, the average spread diameter of all pastes with different SPs had no significant increase or decrease with respect to that of the paste without SP. Therefore, it can be concluded that all SPs investigated in this paper had no significant effect on the workability of the mixture activated by GD Grade sodium silicate powder. This is most likely because all SPs studied in this paper experienced structural changes resulting in loss of their superplasticizing properties when GD Grade sodium silicate powder was used as the activator [32,57].

#### 4.1.2. Compressive Strength

**Figure 2:**
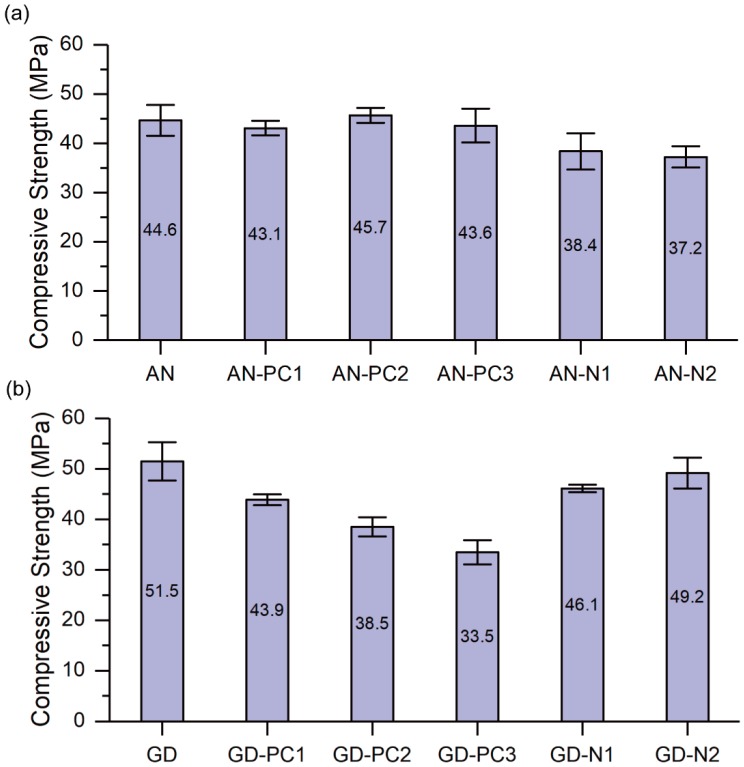
Effect of different SPs on compressive strength of one-part fly ash-slag blended geopolymer pastes activated by (**a**) Anhydrous sodium metasilicate powder, and (**b**) GD Grade sodium silicate powder.

Figure 2 presents the compressive strength of each mixture without and with different SPs. By comparing the pastes without any SP, it was found that the compressive strength of “GD” paste was 16% higher than that of “AN” paste. This is due to the higher modulus of GD Grade sodium silicate (nSiO_2_/nNa_2_O = 2.0) as compared to that of anhydrous sodium metasilicate (nSiO_2_/nNa_2_O = 0.9). Previous studies showed that activator with higher modulus provided more soluble silicate into the geopolymeric system, which promoted the geopolymerisation reaction and resulted in increasing the strength [58,59].

According to Figure 2a, the addition of the PC-based SPs (i.e., PC1, PC2 and PC3) to the one-part geopolymer paste activated by anhydrous sodium metasilicate powder did not have any significant effect on the compressive strength of the paste with reference to the paste without any SP. However, the compressive strengths of the pastes with the N-based SPs (i.e., N1 and N2) were decreased as compared to the paste without any SP. The decrease in compressive strength of “AN-N1” and “AN-N2” pastes was 14% and 17%, respectively with reference to “AN” paste. On the contrary, for the mixtures activated with GD Grade sodium silicate (Figure 2b), the compressive strength of the pastes with the PC-based SPs was remarkably reduced with respect to that of the paste without SP. The reduction in the compressive strengths of “GD-PC1”, “GD-PC2” and “GD-PC3” pastes were 15%, 25% and 35%, respectively with reference to “GD” paste. As can be seen in Figure 2b, the decrease in the compressive strength of the pastes with the N-based SPs was less significant as compared to that of the pastes with PC-based SPs. These results suggested that the variations of compressive strengths obtained in this paper are closely related to the chemical stability of SPs in different alkaline solutions [25,32]. Based on Figure 1 and Figure 2, it can be concluded that PC1 and anhydrous metasilicate powder exhibited good compatibility as the workability of the resultant geopolymer paste was significantly increased, and at the same time no strength reduction was observed.

### 4.2. Effects of Different Retarders

#### 4.2.1. Setting Time

Table 6 presents the initial and final setting times of each mixture without and with different RTs. It should be noted that the setting time measurement was ended if the initial setting time of the paste was beyond 360 min, which is more than enough for all construction applications. As can be seen in this table, the initial and final setting times of “AN” paste were 169 min and 255 min, respectively, while the initial setting time of “GD” paste was beyond 360 min. The anhydrous sodium metasilicate solution has higher pH value (pH = 13.65 at 20 °C) than that of the GD Grade sodium silicate solution (pH = 12.71 at 20 °C). The dissolution rate of amorphous aluminosilicates is higher at high pH environment (pH > 13) which accelerates the geopolymerisation reaction and results in shorter setting time [11,60]. Thus, “AN” paste has shorter setting time than that of “GD” paste due to its faster geopolymerisation reaction.

As can be seen in Table 6, for the pastes activated with anhydrous sodium metasilicate, all RTs studied in this paper significantly increased the initial and final setting times in the range of 102–112% and 79–94%, respectively with respect to the paste without retarder (“AN” paste). Among the RTs investigated in this study, sucrose was the most effective retarder to prolong the setting time. Kusbiantoro et al. [44] also reported the retarding effect of sucrose on ‘two-part’ fly ash-based geopolymer. They envisaged that the HO–C–C = O groups from sucrose were transformed into acid complexes under alkaline environment, which can chelate the calcium, silicon and aluminum ions in the system and delay the formation of calcium silicate hydrates (C–S–H) and calcium aluminate silicate hydrate (C–A–S–H) gels [61]. These gels can be found on the geopolymers containing slag [62,63,64,65,66]. According to Table 6, the initial and final setting times of the paste with borax (“AN-B”) were 15 min and 29 min, respectively shorter than those of the paste with sucrose. The retarding mechanism of borax was studied in detail by Liu et al. [45]. Although the commercial retarder significantly increased the initial and final setting times of the paste as compared to that of the paste without retarder, the final setting time of the paste with the commercial retarder was still 38 min shorter than of the paste with sucrose.

According to Table 6, the initial setting time of all pastes (with and without retarder) activated by GD Grade sodium silicate was more than 360 min, which is acceptable for all construction applications. The final setting time of these pastes was not measured since their initial setting time was beyond 360 min.

#### 4.2.2. Workability

**Figure 3:**
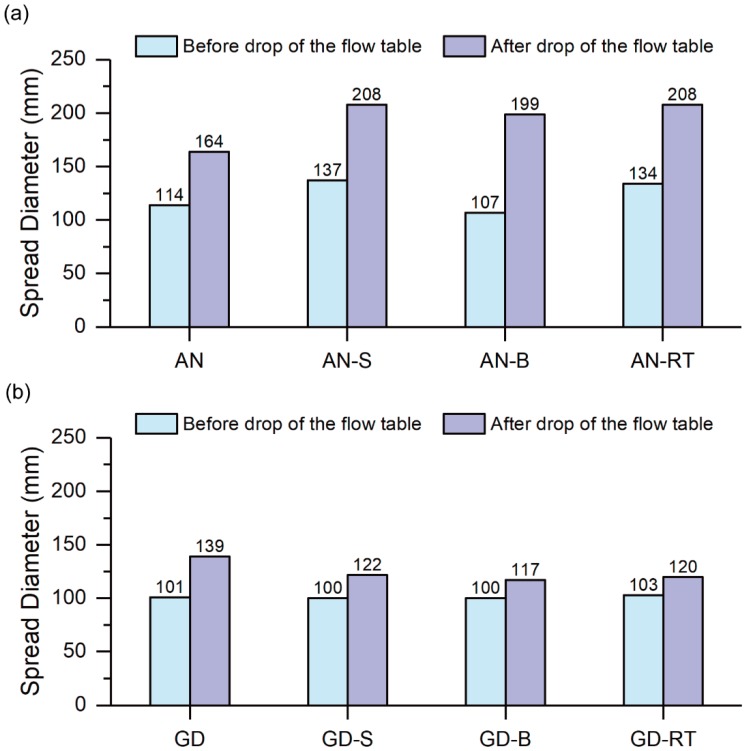
Effect of different RTs on workability of one-part fly ash-slag blended geopolymer pastes activated by (**a**) Anhydrous sodium metasilicate powder, and (**b**) GD Grade sodium silicate powder.

Figure 3 presents the average spread diameter of each mixture without and with different RTs. For the pastes activated by anhydrous sodium metasilicate (Figure 3a), all RTs investigated in this paper improved the spread diameter of the pastes of up to 27% after drop of the flow table. According to Ramachandran [61], most RTs and water reducers are based on the same source materials, two of which are sucrose and borates. Thus, the workability (spread diameter) of the pastes was improved as they can act like plasticizers in this case. Also, in the case of “AN-RT” paste, the additional water from this commercial retarder may also contribute to improving the workability of the paste since this retarder was in liquid form.

On the other hand, in the case of the mixtures activated by GD Grade sodium silicate (Figure 3b), the average spread diameter of the pastes containing RTs before drop of the flow table was comparable to that of the paste without retarder (equal to 100 mm). However, after drop of the flow table the average spread diameter of the pastes with different RTs was 12–16% smaller with respect to that of the paste without retarder. These results indicated that the additional fluidifying effect from the RTs studied in this paper greatly depends on the type of solid activator used. Therefore, it can be said that all types of RTs studied in this paper had negative effect on the workability of the pastes activated by GD Grade sodium silicate. However, an opposite trend was observed for the pastes activated by anhydrous sodium metasilicate. 

#### 4.2.3. Compressive Strength

**Figure 4:**
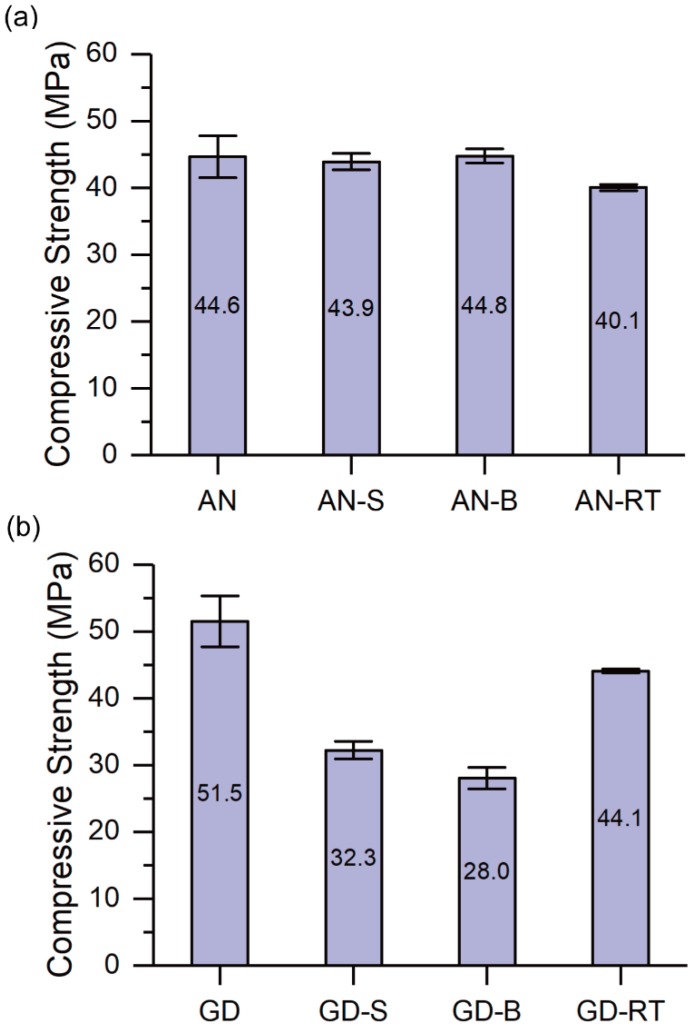
Effect of different RTs on compressive strength of one-part fly ash-slag blended geopolymer pastes activated by (**a**) Anhydrous sodium metasilicate powder, and (**b**) GD Grade sodium silicate powder.

Figure 4 presents the compressive strength of each mixture without and with different RTs. In case of the pastes activated by anhydrous sodium metasilicate (Figure 4a), all RTs had no effect on the compressive strength except the commercial retarder. The compressive strength of the paste with the commercial retarder was 10% lower than that of the paste without retarder. However, as can be seen in Figure 4b, all RTs reduced the compressive strength of the pastes activated by GD Grade sodium silicate. The decrease in the compressive strength of the pastes with sucrose, borax and the commercial retarder was 37%, 46% and 14%, respectively with reference to the paste without retarder. Previous studies also reported a decrease in compressive strength of “two-part” geopolymer when sucrose or borax was used as the retarder [44,45,67].

Based on the results obtained in this paper, the compatibility between activator and retarder has a significant influence on the fresh properties and strength of “one-part” fly ash-slag blended geopolymer paste. As discussed in Section 4.2.1, Section 4.2.2 and Section 4.2.3, when anhydrous sodium metasilicate was used as the activator, all RTs studied in this paper exhibited positive effect on the workability and setting time of the one-part fly ash-slag blended geopolymer mixtures, without decreasing the compressive strength. In addition, sucrose was the most effective retarder among the RTs studied in this paper. However, when GD Grade sodium silicate was used as the activator an opposite trend (i.e., reduction in workability and compressive strength) was observed.

### 4.3. Effects of Combined Admixtures

Based on the results presented in Section 4.1 and Section 4.2, PC1, sucrose and borax were selected to investigate the effect of combined SP and retarder on the one-part fly ash-slag blended geopolymer paste activated by anhydrous sodium metasilicate. Table 7 presents the workability, setting time and compressive strength of the pastes with and without the ‘combined admixtures’. As shown in this table, the average spread diameter of the pastes with the combined admixtures was larger (both before and after drop of the flow table) than that of the paste without any admixture. However, comparison of the workability of the pastes with the combined admixtures (“AN-PC1-S” and “AN-PC1-B”) versus the paste with only the PC1 (“AN-PC1”) showed that the workability of “AN-PC1-S” and “AN-PC1-B” pastes were lower than that of “AN-PC1” paste. This may be due to the competitive absorption between the PC1 and sucrose or borax. It was envisaged that the absorption of the PC1 was reduced due to the presence of sucrose or borax and resulted in reduction in fluidity of the paste. Several authors had reported similar competitive absorption between SP and RT in OPC and sulphoaluminate cement [68,69,70,71].

The setting time of the pastes with the ‘combined admixtures’ was significantly extended compared to the paste without any admixture. The initial and final setting times of “AN-PC1-S” and “AN-PC1-B” pastes were 139–141% and 90–95%, respectively longer with respect to “AN” paste. By comparing these results with that of the pastes with only the retarder (“AN-S” and “AN-B”), the initial setting time of the pastes with the combined admixtures was 14–18% longer than that of the pastes with only the retarder. This may be due to the fluidifying effect of PC1 and/or extra water content from PC1 that prolonged the setting time of the pastes.

As shown in Table 7, the compressive strength of the pastes with the ‘combined admixtures’ was lower than that of the paste without any admixture. The compressive strength of “AN-PC1-S” and “AN-PC1-B” pastes were 16% and 26%, respectively lower than that of “AN” paste. In addition, the compressive strength of “AN-PC1-S” and “AN-PC1-B” pastes was 15–27%, respectively lower than that of the pastes with only the RT (Figure 4). Zhang et al. [69] also reported the compressive strength of sulphoaluminate cement with combined admixtures (combination of a SP with a RT) was decreased. It was reported that the reduction of compressive strength was due to the retarding effect of both retarder and SP, causing some cement grains unreacted. Based on the results presented in Table 7, the combination of PC1 and sucrose would be more effective than the combination with of PC1 and borax as “AN-PC1-S” paste exhibited higher compressive strength, workability and longer setting time as compared to “AN-PC1-B” paste.

## 5. Conclusions

This study investigated the effects of different SPs and RTs on fresh and hardened properties of the one-part fly ash-slag blended geopolymers activated by two different grades of solid sodium silicate, namely anhydrous sodium metasilicate and GD Grade sodium silicate powders. The results revealed that efficiency of different SPs and RTs on the properties of the one-part geopolymers significantly depended on the type of solid activator, as well as the type SP and RT used. Based on the experimental results obtained in this study, the following specific conclusions can be drawn:

1. For the mixture activated by the anhydrous sodium metasilicate powder (with nSiO_2_/nNa_2_O = 0.9), the PC1 (a modified polycarboxylate-based SP) was the most effective SP. The use of PC1 resulted in 72% and 40% increase in the average spread diameters of the paste before and after drop of the flow table, respectively, as compared to the paste without using any SP. At the same time, the use of PC1 did not reduce the compressive strength of the paste.
2. For the mixture activated by GD Grade sodium silicate powder (with nSiO_2_/nNa_2_O = 2.0), none of the investigated SPs were effective in increasing the workability. In addition, the use of naphthalene-based (N-based) SPs resulted in a marginal (up to 10%) reduction in the compressive strength, while the use of polycarboxylate-based (PC-based) SPs resulted in a significant (up to 35%) reduction in the compressive strength, as compared to the paste without using any SP.
3. All investigated RTs significantly increased the initial setting time (up to 112%), final setting time (up to 94%) and workability (up to 27%) of the paste activated by anhydrous sodium metasilicate powder, with no significant effect on the compressive strength. Among the RTs investigated in this study, sucrose was the most effective one in retarding the setting time, with positive effect on the workability and no reduction of the compressive strength.
4. When GD Grade sodium silicate powder was used as the solid activator, the workability and compressive strength of the paste containing the RTs were up to 16% and 46% lower, respectively, as compared to the paste without using any RT.
5. When using the ‘combined admixtures’ (i.e., PC1 in the presence of either sucrose or borax), PC1 was more compatible with sucrose than borax. In other words, the paste containing both PC1 and sucrose exhibited higher compressive strength and workability, along with longer setting time, as compared to the paste containing both PC1 and borax.
6. The compressive strength of the paste containing the ‘combined admixtures’ was up to 26% lower than that of the paste with no admixture. This may be due to the retarding effect of both the retarder and the SP.
7. The workability of the paste containing the ‘combined admixtures’ was up to 32% lower than that of the paste containing only PC1. This may be due to competitive absorption between PC1 and sucrose or borax. It was envisaged that the absorption of PC1 was reduced due to the presence of sucrose or borax and resulted in the in the fluidity reduction of the paste.
8. The initial setting time of the paste containing the ‘combined admixtures’ was up to 18% longer than that of the paste containing only the retarder (either sucrose or borax). This may be due to the fluidifying effect of PC1 and/or extra water content from PC1 that prolonged the setting time.

## Figures and Tables

**Table 1:** Chemical composition of the slag and fly ash (wt%).

Chemical	Component	
	Slag	Fly Ash
SiO_2_	32.76	51.52
Al_2_O_3_	12.37	27.83
Fe_2_O_3_	0.54	11.77
CaO	44.56	2.20
MgO	5.15	1.30
K_2_O	0.33	0.60
Na_2_O	0.22	0.40
MnO	0.15	0.15
TiO_2_	0.51	1.50
P_2_O_5_	0.88	0.73
SO_3_	4.26	0.20
L.O.I. ^1^	0.09	1.80

^1^ Loss on ignition.

**Table 2:** Specifications of different types of sodium silicate powders.

Type of Activator	nSiO_2_/nNa_2_O	SiO_2_^a^ (FR%)	Na_2_O ^a^ (wt%)	H_2_O (wt%)
Anhydrous sodium metasilicate	0.9	46	51	3 ^b^
GD Grade sodium silicate	2.0	54	27	18 ^b^

^a^ Average wt% reported by the supplier; ^b^ Chemically bound water in the powder which is released when dissolved in water.

**Table 3:** Chemical and physical properties of superplasticizers.

SP ID	Commercial Name	Chemical Base	Color	pH	Density (g/cm^3^)
PC1	MasterGlenium SKY 8379	Modified polycarboxylate	Brown	5.9	1.06
PC2	ViscoCrete PC HRF-1	Modified polycarboxylate	Light brown	n.r. ^1^	n.r. ^1^
PC3	ViscoCrete 5-500	Modified polycarboxylate	Clear brown	5.0 ± 1.0	1.07
N1	MasterRheobuild 1000NT	Sodium naphthalene formaldehyte sulphonate	Dark brown	7.0	1.20
N2	Sikament NN	Sodium naphthalene formaldehyte sulphonate	Brown	7. 0 ± 0.5	1.20

Note: The pH and density values are reported by the suppliers at 20 °C; ^1^ not reported by the supplier.

**Table 4:** Physical properties of retarders (RTs).

RT ID	Commercial Name	Physical Form	Color	Density (g/cm^3^)
S	Sucrose RA	Powder	White	1.59
B	Borax Anhydrous Pyrobor	Powder	White	1.12
RT	MasterSet RT 122	Liquid	Dark brown	1.05

Note: The density values are reported by the suppliers at 20 °C.

**Table 5:** The mixture proportions of one-part fly ash-slag blended geopolymer paste.

Mix ID	Geopolymer Precursors	Solid Activator	Water	Superplasticizer	Retarder	W/GP-Solids
AN	1.000	0.100 ^a^	0.400	–	–	0.367
AN-PC1	1.000	0.100 ^a^	0.400	0.010 ^c^	–	0.367
AN-PC2	1.000	0.100 ^a^	0.400	0.010 ^d^	–	0.367
AN-PC3	1.000	0.100 ^a^	0.400	0.010 ^e^	–	0.367
AN-N1	1.000	0.100 ^a^	0.400	0.010 ^f^	–	0.367
AN-N2	1.000	0.100 ^a^	0.400	0.010 ^g^	–	0.367
AN-S	1.000	0.100 ^a^	0.400	–	0.010 ^h^	0.367
AN-B	1.000	0.100 ^a^	0.400	–	0.010 ^i^	0.367
AN-RT	1.000	0.100 ^a^	0.400	–	0.010 ^j^	0.367
GD	1.000	0.100 ^b^	0.379	–	–	0.367
GD-PC1	1.000	0.100 ^b^	0.379	0.010 ^c^	–	0.367
GD-PC2	1.000	0.100 ^b^	0.379	0.010 ^d^	–	0.367
GD-PC3	1.000	0.100 ^b^	0.379	0.010 ^e^	–	0.367
GD-N1	1.000	0.100 ^b^	0.379	0.010 ^f^	–	0.367
GD-N2	1.000	0.100 ^b^	0.379	0.010 ^g^	–	0.367
GD-S	1.000	0.100 ^b^	0.379	–	0.010 ^h^	0.367
GD-B	1.000	0.100 ^b^	0.379	–	0.010 ^i^	0.367
GD-RT	1.000	0.100 ^b^	0.379	–	0.010 ^j^	0.367

Note: All numbers are mass ratios of the geopolymer precursors (fly ash + slag) weight; ^a^ Anhydrous sodium metasilicate powder; ^b^ GD Grade sodium silicate powder; ^c^ PC1 superplasticizer; ^d^ PC2 superplasticizer; ^e^ PC3 superplasticizer; ^f^ N1 superplasticizer; ^g^ N2 superplasticizer; ^h^ Sucrose; ^i^ Borax; ^j^ RT retarder.

**Table 6:** Effect of different RTs on setting time of one-part fly ash-slag blended geopolymer pastes activated by different activators.

Mix ID	Setting Time (min)	
	Initial	Final
AN	169	255
AN-S	357	495
AN-B	342	466
AN-RT	359	457
GD	>360	– *
GD-S	>360	– *
GD-B	>360	– *
GD-RT	>360	– *

* Not measured since the initial setting time was more than 360 min.

**Table 7:** Effect of combined admixtures on one-part fly ash-slag blended geopolymer paste.

Mix ID	Compressive Strength (MPa)	Workability		Setting Time	
		Before Drop of Flow Table (mm)	After Drop of Flow Table (mm)	Initial (min)	Final (min)
AN	44.6 ± 3.1	114	164	169	255
AN-PC1-S ^a^	37.5 ± 2.8	159	205	407	497
AN-PC1-B ^b^	32.8 ± 3.7	133	206	404	484

^a^ Geopolymer mixture with combination of PC1 and sucrose; ^b^ Geopolymer mixture with combination of PC1 and borax.
